# The Effect of Surface Wave Propagation on Neural Responses to Vibration in Primate Glabrous Skin

**DOI:** 10.1371/journal.pone.0031203

**Published:** 2012-02-13

**Authors:** Louise R. Manfredi, Andrew T. Baker, Damian O. Elias, John F. Dammann, Mark C. Zielinski, Vicky S. Polashock, Sliman J. Bensmaia

**Affiliations:** 1 Department of Organismal Biology and Anatomy, University of Chicago, Chicago, Illinois, United States of America; 2 Kimberly-Clark Corporation, Roswell, Georgia, United States of America; 3 Department of Environmental Science, Policy, and Management, University of California, Berkeley, Berkeley, California, United States of America; Instituto de Neurociencias de Alicante UMH-CSIC, Spain

## Abstract

Because tactile perception relies on the response of large populations of receptors distributed across the skin, we seek to characterize how a mechanical deformation of the skin at one location affects the skin at another. To this end, we introduce a novel non-contact method to characterize the surface waves produced in the skin under a variety of stimulation conditions. Specifically, we deliver vibrations to the fingertip using a vibratory actuator and measure, using a laser Doppler vibrometer, the surface waves at different distances from the locus of stimulation. First, we show that a vibration applied to the fingertip travels *at least* the length of the finger and that the rate at which it decays is dependent on stimulus frequency. Furthermore, the resonant frequency of the skin matches the frequency at which a subpopulation of afferents, namely Pacinian afferents, is most sensitive. We show that this skin resonance can lead to a two-fold increase in the strength of the response of a simulated afferent population. Second, the rate at which vibrations propagate across the skin is dependent on the stimulus frequency and plateaus at 7 m/s. The resulting delay in neural activation across locations does not substantially blur the temporal patterning in simulated populations of afferents for frequencies less than 200 Hz, which has important implications about how vibratory frequency is encoded in the responses of somatosensory neurons. Third, we show that, despite the dependence of decay rate and propagation speed on frequency, the waveform of a complex vibration is well preserved as it travels across the skin. Our results suggest, then, that the propagation of surface waves promotes the encoding of spectrally complex vibrations as the entire neural population is exposed to essentially the same stimulus. We also discuss the implications of our results for biomechanical models of the skin.

## Introduction

When we run our fingers across a textured surface, small vibrations are produced in the skin. These vibrations are spectrally complex and depend on complex interactions between skin and surface [1], [2]. For instance, satin elicits different vibrations than does silk, and it is based on these differences that we are able to distinguish one from the other. Texture-elicited vibrations are transduced by specialized receptors embedded in the skin that convey information about the microgeometry of the surface [1]–[4], namely Pacinian corpuscles. When these receptors and their associated afferent fibers (PC fibers) are desensitized, the perception of surface microgeometry is severely impaired or abolished [5]. That the perception of texture relies on the analysis of spectrally complex oscillations has led to the suggestion that it may be analogous to the perception of auditory timbre [2], [6]. In addition to their role in texture perception, skin vibrations may also play a role in the perception of distal events. For example, when we use tools, vibrations transmitted through the tool convey information about events at the distal end of the tool or about the properties of objects contacting the tool [7]–[10]. Importantly, vibrations travel across the skin, which is thought to play an important sensory role [11]–[13]. Indeed, these traveling waves recruit a larger population of PC afferents than would be activated if it were restricted to PC fibers with receptive fields near the locus of contact [13]. However, the decay of these waves has not been quantitatively characterized in the glabrous skin of the hand, so the actual size of the PC population activated by a vibratory stimulus or textured surface is unknown. Traveling waves may thus amplify the signal. However, they may also serve to alter it: To the extent that the waveform of a vibration becomes distorted as it propagates across the skin, spatially displaced receptors may experience a different stimulus. Spatially displaced afferents would then carry discrepant information about the stimulus and the traveling waves may thus interfere with the veridical perception of the stimulus.

## Results and Discussion

**Figure 1:**
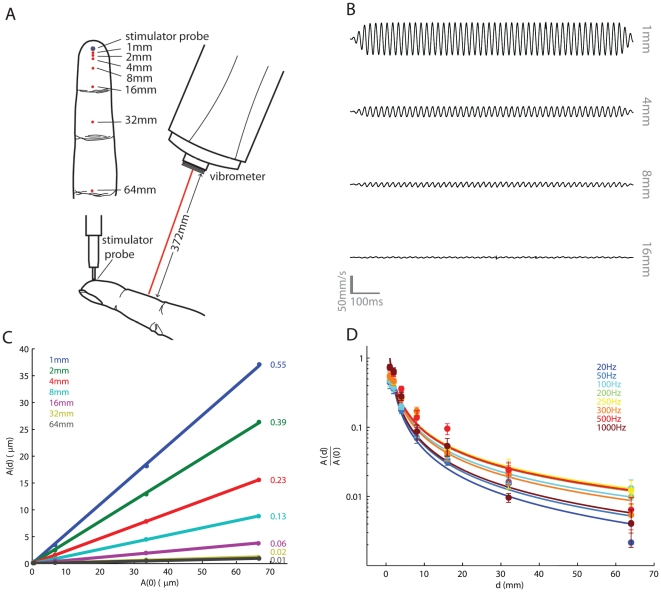
Measurement of skin vibrations. A. Experimental set-up. Vibrations are delivered to the fingertip through a probe while movements of the skin are measured with a laser Doppler vibrometer at various distances from the locus of stimulation (see top left inset). B. Traces from the LDV for a 50-Hz stimulus recorded at 4 distances away from the locus of stimulation. C. Amplitude of the vibrations, measured at distance *d* from the locus of stimulation as a function of the amplitude of the vibrations delivered (*f* = 200 Hz), computed from sample data from one participant (numbers to the right denote the slope of the corresponding function). D. Ratio of the measured amplitude to the delivered amplitude as a function of distance, averaged across participants (error bars denote the SEM). As expected, surfaces waves decay as they travel away from the stimulator, and the relationship can be approximated using Equation 1. However, the rate of decay depends on the vibratory frequency.

Here, we use a laser Doppler vibrometer (LDV) to measure the speed and decay of surface waves produced on the skin using a vibration exciter. Specifically, we deliver to the fingertip a vibration of known frequency (ranging from 50 to 1000 Hz) and amplitude (ranging from 0.1 to 200 µm), spanning the range of every day tactile experience, and measure the surface waves at various distances (ranging from 1 to 64 mm) from the locus of stimulation (Figure 1A,B). To measure decay, we compute the slope of the function relating the measured amplitude, *A(d)*, to stimulus amplitude, *A(0)*, as a function of the distance *d* from the locus of stimulation. This slope gauges the ratio between the amplitude of the surface wave and that of the stimulus: a slope of 1 indicates no decay; a slope of 0 indicates complete decay (Figure 1C).

As might be expected, the amplitude of surface waves decreases as they travel away from the locus of stimulation. The decay of the surface waves can be described using the expression:
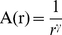
(1)where *γ* denotes the rate of decay, with faster decay rates denoted by higher *γ*'s. We verified that the power function provided a significantly better fit than did an exponential one, which has been found to describe decay rates on the arm [14]. To this end, we computed the correlation between the predicted and measured slopes across locations at each frequency and for each participant. We then computed the Fisher's Z-transform of each correlation (to stabilize the variance across correlation values) and performed a paired t-test on (Fisher's Z-transformed) correlations corresponding to each frequency and participant. We found that the power function provided a significantly better fit than did the exponential function (*t*(39) = 3.64, *p*<0.001).

As shown in Figure 1D, skin vibrations travel over a wide range. Indeed, vibrations can be detected 64 mm away from contactor tip. Given the exquisite sensitivity of Pacinian afferents, even these residual vibrations are liable to produce a response provided A(0) is of sufficient intensity. For example, at 250 Hz, the absolute threshold of Pacinian fibers is on the order of 100 nm [13], [15]; thus, a 10-µm, 250-Hz stimulus would activate a Pacinian afferent located 6 cm from the locus of stimulation. Given these results, the large size of Pacinian receptive fields, which typically span one or more digits or the entire palm of the hand, can be accounted for based on the distance travelled by surface waves.

Furthermore, the decay of the vibrations is dependent on the frequency of the stimulus. Specifically, vibrations decay more rapidly at low and high frequencies (e.g., 20 and 1000 Hz) than they do at intermediate frequencies, with decay being slowest at 200 and 250 Hz (with mean *γ* = 1.08 and 1.1, respectively) and highest at 20 and 1000 Hz (with mean *γ* = 1.3 and 1.27, respectively). The effect of frequency on decay rate was statistically significant (repeated measures ANOVA, F(7,32) = 3.53, p<0.01). The shape of the function relating *γ* to frequency suggests that the skin resonates at 200–250 Hz, which closely matches the peak sensitivity of Pacinian afferents [13], [16]. Note that the frequency sensitivity profile of PC afferents cannot be explained solely on the basis of skin resonance: The absolute threshold of PC afferents, lowest at 250–300 Hz [13], [16], is typically determined by placing the stimulating probe at the point of maximum sensitivity of the afferent and is thus little affected by surface waves. Furthermore, similar PC spectral sensitivity functions have also been measured in afferents excised from cat mesentery, thereby removing any influence of the skin on sensitivity [17]. Rather, the match between skin resonance and PC sensitivity suggests that the biomechanical properties of the skin and the response properties of PC afferents may have co-evolved to optimize sensitivity to vibrations with frequencies around 200–300 Hz. Indeed, the spatial period of fingerprint skin may also be optimized for maximum PC activation during natural haptic exploration of surfaces [18].

### Effect of resonance on the strength of the afferent response

**Figure 2:**
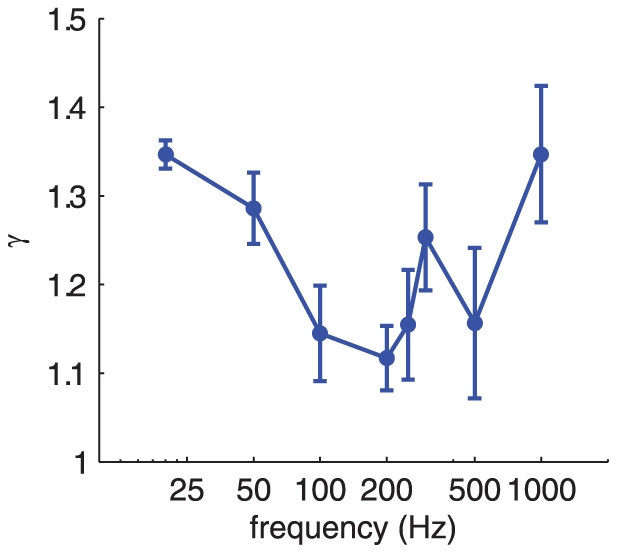
Decay exponent as a function of frequency across participants (error bars denote SEM). The decay exponent is lowest (and so decay is slowest) at around 200–250 Hz, at which Pacinian afferents are most sensitive.

**Figure 3:**
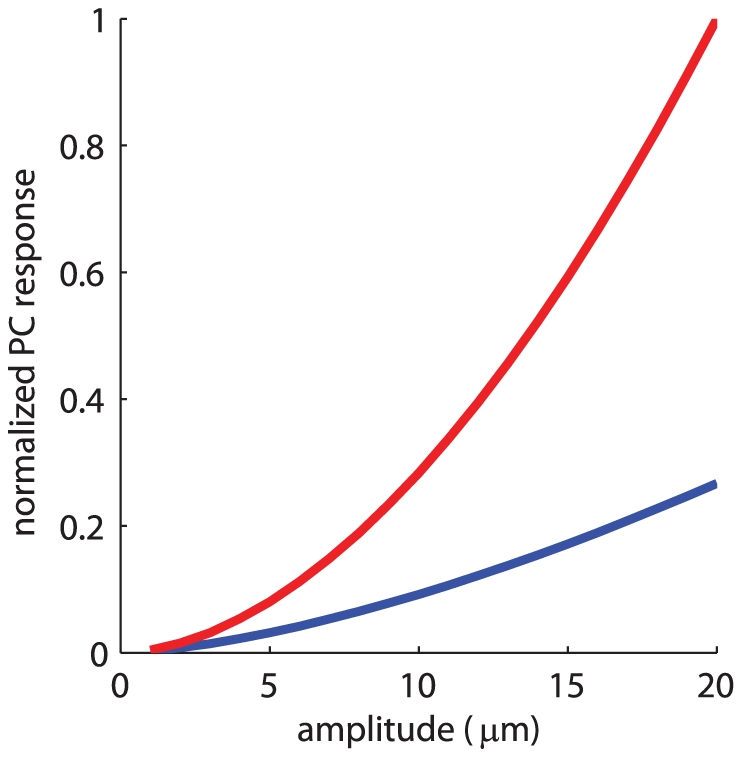
Estimated PC population firing rate or number of active PC fibers (normalized by their respective maxima) as a function of amplitude for a 200-Hz stimulus, using an γ of 1.3 (blue) and 1.1 (red). As can be seen, the firing rate and active population is almost twice as large for the latter than it is for the former for large amplitudes. Note that, given that the stimulus frequency remains constant, the number of active fibers and the overall firing rate of the population are linearly related.

While the effect of frequency on decay rate seems to be relatively minor upon inspection of the exponents (Figure 2), the relatively small numerical difference in exponent may have a substantial effect on the magnitude of the surface waves, particularly at locations far removed from the locus of stimulation. At 64 mm, for example, ratios (A(d)/A(0)) spanned the range from 0.4 to 1.2% and, at 16 mm, from 25 to 53%; these differences in decay rate are therefore potentially behaviorally relevant effects, especially considering that these differences are integrated over the skin surface. We approximated the effect of these differences in decay rate on the strength of the PC response by comparing the firing rates evoked in a simulated population of PC afferents by a 200-Hz stimulus (estimated using measured sensitivity functions [13], [19]) using exponents of 1.3 and 1.1 (measured at 20 and 200 Hz, respectively). We found that the slower decay rate at 200 Hz could have a substantial effect on the magnitude of the population response, particularly for larger vibrations (Figure 3). Indeed, for a 200-Hz, 20-µm stimulus, the estimated response was twice as large, as was the estimated number of active PC fibers, with an exponent of 1.1 than with an exponent of 1.3. Thus, skin resonance substantially increases the strength of the afferent response to skin vibrations.

### Effect of surface waves on the temporal patterning in the responses of afferent populations

**Figure 4:**
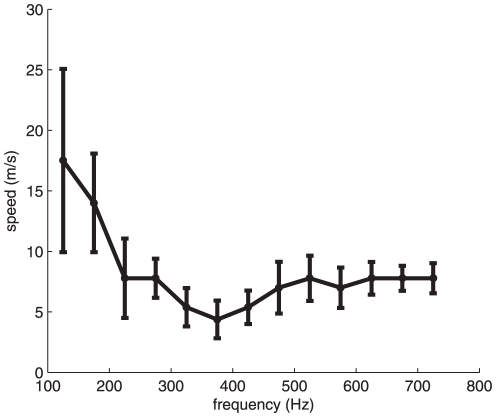
Propagation speed as a function of frequency for band-pass noise stimuli, filtered using narrow-band filters, averaged across participants. The values on the x-axis represent the center frequency of the filters (each 100 Hz wide). Error bars denote the SEM. Surprisingly, low-frequency components (<200 Hz) seem to travel faster than high frequency components.

We measured the speed of propagation of surface waves by measuring the time it took the waves to travel from the locus of stimulation to the three measurement points (1, 8, and 16 mm). We found that propagation speed was highest at low frequencies, peaking around 17 m/s, and decreased as a function of frequency, leveling off at around 7 m/s (Figure 4). These velocities are commensurate with previous measurements on the hand and somewhat higher than those on the forearm [14], [20].

**Figure 5:**
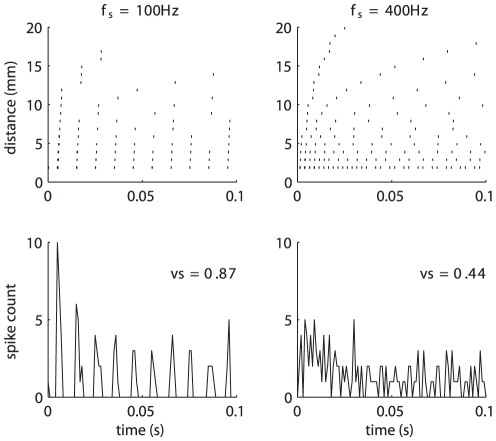
Response of a population of simulated PC afferents to a 100-Hz (left) and a 400-Hz (right) sinusoid applied to the skin. The PC population exhibits a highly temporally patterned response to the 100-Hz but not the 400-Hz stimulus as reflected in the vector strengths of 0.87 and 0.44, respectively. The lack of temporal patterning at 400 Hz is due in part to the delay in the response for fibers whose receptive fields are progressively further from the locus of stimulation.

Mechanoreceptive afferents produce highly repeatable and temporally patterned responses to periodic vibrations delivered to the skin. For sinusoidal stimuli, the interval between bursts of action potentials is proportional to the period of the stimulus [16] and temporal patterning is observed in afferent responses to polyharmonic and even to noise stimuli [13]. This temporal patterning is thought to convey information about the frequency of skin oscillations [16]. Neurons in somatosensory cortex also produce entrained responses to sinusoidal stimulation, but this entrainment is only observed up to frequencies of about 200 Hz, whereas PC responses can become entrained up to about 800–1000 Hz. One possibility is that the loss of entrainment for high-frequency stimuli in cortex is due to the fact that these reflect the combined responses of spatially scattered afferents. Indeed, individual S1 neurons receive input from multiple afferents that are displaced from one another [21]. As a result, a localized stimulus is liable to impinge upon different afferents (whose responses ultimately converge on a single neuron) at different times, causing a blurring in the temporal patterning of their combined response: Afferents close to the locus of stimulation will tend to fire sooner within each stimulus cycle than those further away. We can assess the degree to which this is the case by simulating the response of a distributed population of PC afferents to a stimulus, using the measured speed and decay of the waves and a model of mechanotransduction for this afferent population [22]. Figure 5 shows the responses of such a population to two stimuli at different frequencies (100 and 400 Hz). As can be seen, the pooled response from the population (shown in the peristimulus time histogram) exhibits strong temporal patterning at 100 Hz and little to no patterning at 400 Hz. Thus, a neuron that receives convergent input from a distributed PC population will exhibit temporal patterning to a 100-Hz stimulus but not to a 400-Hz one.

**Figure 6:**
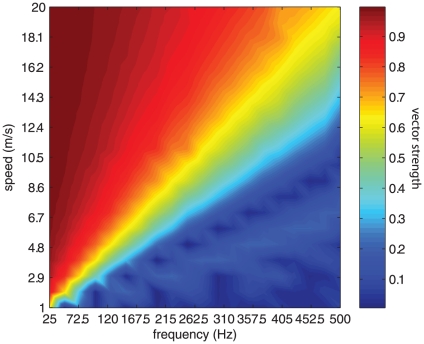
Entrainment (measured using vector strength) of a population of PC afferents in response to stimuli as a function of the frequency of the stimulus and of the propagation speed of the surface waves. Low frequencies are less susceptible to temporal blur and higher speeds tend to cause less blur.

We can examine the effect of blurring as a function of propagation speed and of stimulus frequency by simulating populations of afferents to stimuli varying in frequency while also assuming different speeds. As can be seen in Figure 6, surface waves set an upper bound on the frequencies that can be represented temporally in the firing of mechanoreceptive afferent populations (in this case, PC afferents since they are the only population that responds at these high frequencies), and this upper bound is dependent on propagation speed. We find that the faster the surface waves, the less they affect population entrainment. Furthermore, the lower the stimulus frequency, the less entrainment in the afferent response is susceptible to this blurring. Given the speed at which surface waves travel (leveling off at around 7 m/s on average), entrainment can be observed at the population level up to frequencies of about 200 Hz (note that the faster speeds at lower frequency have no impact on entrainment). Beyond this frequency, the temporal blurring due to differences in stimulus arrival time begins to obscure the entrainment present in the responses of individual afferents. Entrainment in the responses of S1 neurons begins to drop steeply at around 50 Hz [21], [23], so surface waves do not account for the lack of high-frequency entrainment observed in cortex.

### Waveform distortion during propagation

**Figure 7:**
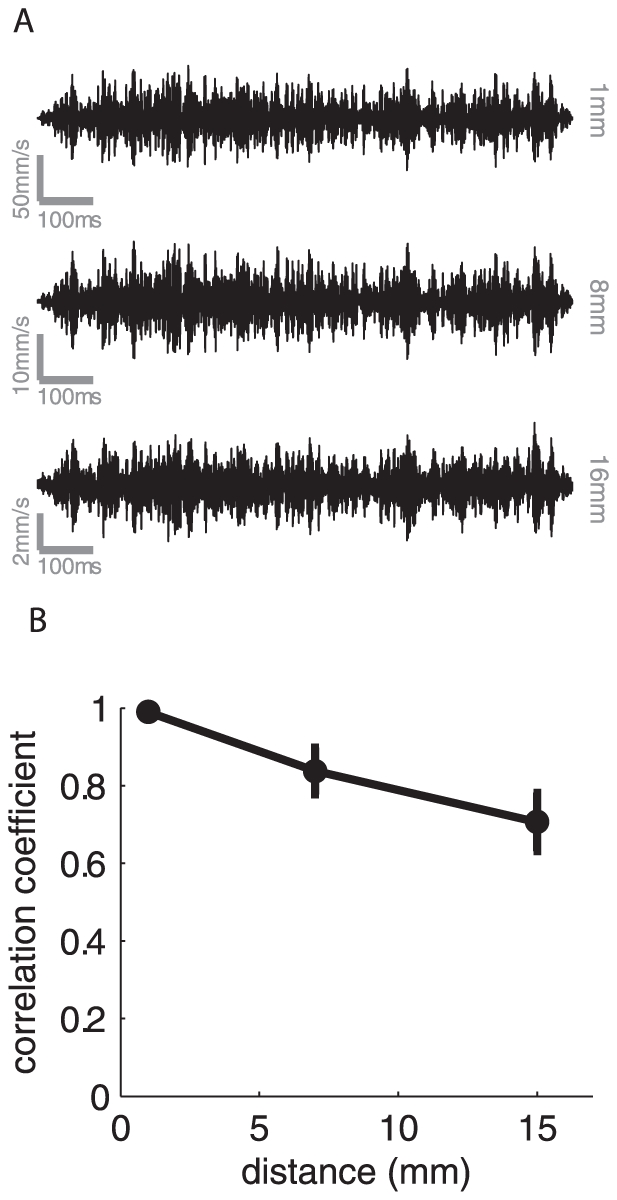
Waveform distortion as a function of distance from the locus of stimulation. A. Traces of traveling waves produced by a noise stimulus (low- and high- frequency cut-offs of 300 and 600 Hz, respectively) measured 1, 8 and 16 mm away from the locus of stimulation. B. Correlation between the stimulus applied to the skin (measured using the accelerometer on the vibration exciter) and the stimulus measured at various distances from the locus of stimulation, averaged across participants (error bars denote SEM). Stimuli consisted of band-pass noise with various low- and high-frequency cut offs. We find that the waveform is, on average, well preserved as it travels along the finger.

We have shown that the rate of decay is dependent on the stimulus frequency, as is the speed of propagation of surface waves. That both of these factors are frequency dependent will contribute to a progressive distortion of the waveform as it travels away from the locus of stimulation. The resonance (or frequency-dependence of the decay rate) will result in the compression of certain components relative to others, while the differential speed will change the phase relationships between components. The frequency dependence of propagation speed will be particularly pronounced for stimuli that comprise low frequency components (<200 Hz) as the slope relating propagation speed to frequency is particularly steep at those frequencies. We can assess the extent to which these two factors result in an overall distortion of the waveform by computing the correlation between the waveform delivered and that measured at various distances from the locus of stimulation (corrected for the propagation lag, computed from the cross correlation). As shown in Figure 7, the average distortion is minimal. The mean correlation between the delivered and measured waveforms 16 mm away from the locus of stimulation is 0.7. Thus, the structure of the stimulus is preserved in the surface waves as they propagate away from the locus of stimulation. Thus, surface waves enhance the strength of the response to a stimulus without distorting its structure.

### Implications for biomechanical models of the skin

A variety of models have been proposed to describe the behavior of the skin under different static and dynamic stimulation conditions. These models general fall under two classes: finite element models [24]–[26] and analytical models [27]–[29]. In the present study, we provide precise quantitative measurements of the propagation of surface waves across the skin. We can test whether the measured behavior of the skin is accounted for by these existing models. Any discrepancy between model and data can then lead to refinements in the models. Our measurements have at least three important implications for models of skin mechanics:

First, the speed at which surface waves propagate across human glabrous skin decreases with frequency over a range of frequencies (as shown in Figure 4). Models of skin mechanics predict that propagation speed will increase with frequency provided that the viscosity of the skin is independent of frequency [30]. Our measurements of propagation speed suggest that, in fact, viscosity decreases as a function of frequency, as has been previously found [20]. Indeed, the propagation speed of *shear* waves has been found to decrease over the range of about 400 to 800 Hz, a behavior attributed to shear thinning of the viscous component of the tissue. The shear thinning behavior of skin layers has been incorporated into mathematical models of the hand in order to improve the predictive power of finite element models describing vibrations in the soft tissues of the body [31]. We were unable to measure the speed of propagation of surface waves in the finite element model of the skin that we implemented because the waves were too distorted to be able to use our analysis based on cross-correlation (see below). However, our measurements can be used to verify that the implementation of shear thinning in these models correctly predicts the propagation speed of surface waves.

**Figure 8:**
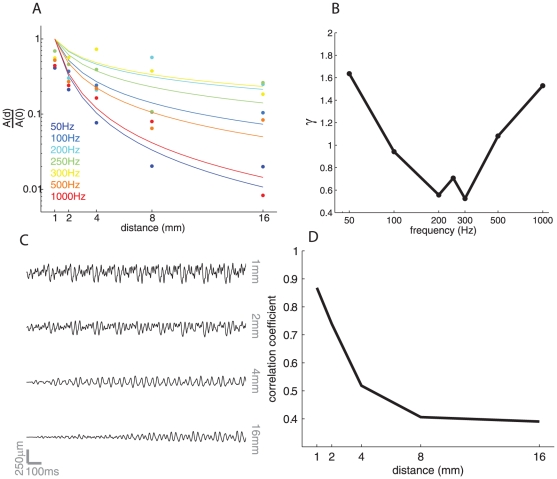
Results from finite element analysis. A. Decay of the traveling waves as a function of distance from the locus of stimulation (at *d* = 0) (Dots are measured points, traces are fitted functions). The results are qualitatively similar to those obtained using the vibrometry data, but the predicted decay is lower at intermediate frequencies (200–300 Hz) than the observed decay (Figure 1). Note that the propagating waves become severely distorted as they travel away from the locus of stimulation, so the measured amplitudes are not a smooth function of distance as they are in the vibrometry recordings. B. Decay exponent, γ, as a function of frequency. The decay exponent is lowest (and so decay is slowest) at around 200–300 Hz, but the modulation as a function of frequency is overestimated by the FEA. C. Traces of simulated traveling waves measured at four locations away from the locus of stimulation. As the wave travels away from the locus of stimulation, the waveform gets rapidly distorted. D. Correlation between the actual waveform delivered by the (virtual) motor and the waveform as it travels down the figure. The rate at which the waveform gets distorted based on the FEA prediction is much more rapid than that observed in the finger (Figure 7).

Second, in the finger, the rate of decay of surface waves is frequency dependent. We implemented a state-of-the art finite element model of the human finger [31] to ascertain whether it exhibited this property. Specifically, we stimulated the virtual finger with sinusoids at various frequencies and measured the amplitude of surfaces waves at various locations away from a virtual probe. We found that the decay followed the relationship described in Equation 1 (Figure 8A, B). Furthermore, the decay of the traveling waves in the virtual finger was dependent on stimulus frequency. However, the strength of the skin resonance (around 300 Hz) was strongly overestimated by the model (compare Figures 2, 3 to Figures 8A, B). Indeed, at 200–300 Hz, the exponent was around 0.5–0.6, which implies a substantially lower decay rate than what is actually observed; at lower and higher frequencies, the rate of decay is higher than that observed.

Third, waveforms undergo little distortion as they propagate across human skin. We measured the traveling wave produced by a polyharmonic stimulus in a virtual finger using finite element analysis and found that its waveform became rapidly distorted as it traveled away from the virtual probe (Figure 8C, D). Finite element models of the skin predict that traveling waves reflect off the bone; reflection, coupled with the strong resonance of the model finger at 300 Hz (Figure 8A, B), will substantially distort surface waves traveling away from the locus of stimulation.

Our results suggest, then, that finite element models of the skin should be refined so that the surface waves decay at the appropriate rate and are less distorted as they propagate across the skin. Also, the propagation speed predicted by these models should be investigated and compared to that measured here.

### Conclusions

Using laser Doppler vibrometry, a non-contact method to measure surface waves elicited in the skin during mechanical stimulation, we have shown that these waves travel long distances across the skin. First, we find that these waves substantially amplify the neural response to the stimulus; in other words, without the propagation of these waves across the skin surface, the response to 200–300 Hz vibrations applied to the skin would be radically reduced. In fact, the effect of a wave propagation on vibratory perception has been demonstrated by showing that vibrations are less detectable when they are delivered with a ring surrounding the vibrating probe (and thus preventing the spread of vibrations) than without [32]. Furthermore, because the rate of decay is dependent on frequency, this amplification of the neural response is also frequency-dependent. Interestingly, the resonant frequency of the skin matches the frequency of maximum sensitivity of Pacinian fibers, which are the most sensitive afferent population to vibration over a wide range of frequencies. Second, we show that surface waves result in a reduction of the temporal patterning in the response of afferent populations, particularly at frequencies over 200 Hz, but the degree of temporal blurring is relatively small compared to that observed in the response of S1 neurons. Third, despite these two factors, the structure of the waveform is well preserved in the form of the surface waves, suggesting that surface waves should enhance the perception of simple and complex skin oscillations. Finally, we discuss three results that will lead to refinements of existing biomechanical models of the skin, namely the decrease of propagation speed with frequency, the specific frequency-dependence of decay rate, and the preservation of the stimulus waveform.

## Materials and Methods

### Calibration and sensitivity analysis

Calibration and sensitivity analysis were conducted by measuring, using a laser Doppler Vibrometer (Polytec OFV-3001 with OFV 311 sensor head, Polytec, Inc., Irvine, CA), known vibrations, generated using a vibration exciter (Mini Shaker 4810, Brüel & Kjær, Skodsborvey, Denmark), at various frequencies, ranging from 5 to 1000 Hz, and amplitudes, ranging from 0.1 to 650 µm. In these experiments, the laser beam impinged directly upon the vibrating probe at a 90° angle (unless otherwise specified) to record from the normal plane of the vibrating probe. The vibration exciter was instrumented with an accelerometer as an independently calibrated device to measure the vibrations. We could then compare the LDV and accelerometer output to assess the extent to which the LDV faithfully recorded the induced vibrations. Specifically, we performed a Fourier analysis of the accelerometer and LDV output. The acceleration and velocity values in the FFT derived from the accelerometer and LDV outputs, respectively, were converted to displacement by dividing the accelerations by (2πf)^2^ and the velocity by 2πf. The amplitude at the stimulus frequency estimated from the LDV was then plotted against that measured from the LDV. The degree to which these fell on the unity line gauged the extent to which the LDV conveys a faithful representation of the vibration at each frequency and amplitude.

First, we tested the LDV at its three sensitivity settings with peak speeds at 20, 100, and 500 mm/s. The low pass filter on the unit was set to 5 kHz, and the high pass filter was disabled. The distance between the probe on the Mini Shaker and the LDV lens housing was 372 mm, stated to be in the optimal stand-off range. Second, we assessed the extent to which vibration measurements were sensitive to the stand-off range by measuring known vibrations at multiple stand-off ranges (234, 303 and 372 mm). Finally, we recorded vibrometry data from different angles of incidence to assess the effect of this measurement parameter on the recorded signal.

At the 20-mm/s peak velocity setting, the LDV failed to measure higher amplitude vibrations across a wide range of frequencies, whereas at 100 and 500 mm/s, most vibrations were recorded accurately (the correlations between the accelerometer- and LDV-measured vibrations were 0.97 and 1.00 at 100 and 500 mm/s, respectively).

Three stand-off distances were used to measure a subset of vibrations from the Mini Shaker. For each of these measurements, the beam was adjusted until the focus indicator on the LDV control panel was at maximum strength. When the beam was fully focused, the stand-off distance did not affect the faithful recording of the Mini Shaker's vibrations (correlations were 0.94, 0.95 and 0.95 at distances of 234, 303, and 372 mm, respectively).

Manipulation of angle of incidence resulted in a multiplicative shift in measured amplitudes. We could correct for this shift by multiplying the measured amplitude by the following quantity:
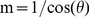
(2)where *θ* is the angle of incidence.

Thus, the LDV was able to measure vibrations down to the lowest tested amplitude (0.1 µm), and was relatively insensitive to stand-off distance and angle of incidence.

### Skin surface vibrations

In previous studies, surface wave propagation and decay has been measured using an apparatus that makes contact with the skin, namely a skin-mounted accelerometer [14], or a pick-up transducer fashioned from a phonograph cartridge and a stylus [20]. These measurement devices may thus affect the vibrations themselves. Laser Doppler vibrometry allows us to record the vibrations elicited in the skin without disrupting them.

To stimulate and record vibrations from the surface of the finger pad, a new experimental rig was designed such that the Mini Shaker and the arm holder were fixed to separate frames. Foam padding was also used between the frame and the experimental table (made of granite) to further reduce the travel of vibrations through the experimental apparatus and framework. Stimulation was applied to the left index fingerpad. The stimulating probe (2 mm in diameter and driven by the Mini Shaker) was placed ¼ of the distance between the fingertip and the distal interphalangeal joint (across participants ranging from 5.5 to 6.5 mm from the tip). Measurements were then made at 1, 2, 4, 8, 16, 32 and 64 mm away from the locus of stimulation (measured from the edge of the stimulating probe). To ensure maximum reflectivity (and maximum signal strength), ultra-thin white-out tape was applied to the skin on the crosshairs in small squares. We verified in preliminary measurements that the white-out tape had no impact on the surface wave mechanics.

The fingernail was then glued to the finger-holder to eliminate finger movements and the probe tip was glued to the finger so that the skin, when stimulated, would oscillate around its resting position. Note that, if the skin is not glued to the finger, then it does not follow the probe on the retraction portion of each cycle, and so does not follow a sinusoidal trajectory [33]. The laser was focused onto each location on the skin successively (at a measured distance from the locus of stimulation, on a patch of skin covered with white-out) and vibrations were delivered to the fingertip. Vibrations ranged in frequency from 20 to 1000 Hz and in amplitude from 0.1 to 200 µm. Each stimulus was presented five times at each location for each of five participants (2 m, 3f), ranging in age from 22 to 37 years. The Human Subjects Institutional Review Board of University of Chicago approved all procedures used in this study. Written consent was obtained from all participants.

For some of the vibrations, the power of the measured vibrations was distributed across harmonics, so the root mean square (RMS) of the LDV signal was computed as a more robust measure of amplitude than the amplitude at the peak frequency, computed from the FFT. We then computed the regression between the LDV-measured rms amplitude and the accelerometer rms amplitude (after both were converted to displacements from velocity and acceleration, respectively). The slope of the regression was a gauge of the ratio between measured vibration and the vibration delivered at the fingertip.

### Measuring the speed of propagation

To measure the speed of propagation, we used band-pass noise stimuli rather than sinusoids because the cross-correlation of two sinusoids (of the same frequency) has a strong oscillatory component, which makes it difficult to accurately identify its peak. To assess the dependence of speed on frequency, we filtered each noise trace using a narrow band-pass (50 Hz) with centers ranging from 100 to 750 Hz (using the zero-phase digital filtering function *filtfilt* in MATLAB, Mathworks, Inc., Natick, MA). We then computed the cross-correlation between the band-pass filtered vibration delivered using the vibration exciter (i.e., the output of the accelerometer) and the band-passed filtered vibration measured at a distance *d* from the locus of stimulation. We then identified the lag *δt* of the peak of the cross-correlation for each stimulus and computed the speed *v_f_* as:
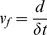
(3)where *f* is the center of the band-pass. The values reported in Figure 4 are the speeds, averaged across stimuli and subjects, at each band-pass (the abscissa corresponds to the center of the band-pass).

### Reconstructing the strength of the PC population response

We used the same approach as that used in a previous study to estimate the firing rate evoked in a population of PC afferents by a 200-Hz stimulus varying in amplitude from 0.1 to 20 µm. We have previously shown that the population response of afferents to a stimulus of amplitude *A* can be very accurately estimated using a rectified logarithmic function:
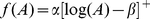
(4)where α and β are the slope and absolute threshold (both dependent on stimulus frequency) and the + sign denotes half-wave rectification as firing rates cannot be negative [13]. In the present study, we show that the decay of surface waves as a function of distance can be approximated using a power function (Equation 1). Thus, the firing rate of afferents located at a distance *r* from the point of stimulation is given by:
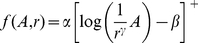
(5)Thus, the firing rate, F_pop_, in a population of afferents can be estimated using the following expression:
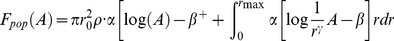
(6)Where *r_0_* is the radius of the stimulating probe, *ρ* is the innervation density of PC afferents (0.2/mm^2^) [34], and *r_max_* is the distance away from the locus of stimulation at which afferents no longer respond, given by:
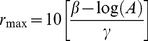
(7)The first term in Equation 6 denotes the response of afferents located under the stimulating probe, and the second term, that of the remaining population of afferents. This approach is described in greater detail in a previous paper [13].

To estimate the size of the activated population, we represented the probability of a given afferent being activated by a stimulus of amplitude *A* using a standard sigmoid:
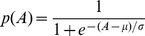
(8)where *μ* and *σ* represent the (known) mean and slope of the sigmoid (estimated in a previous study [13]). Using the logic sketched out above, the probability of an afferent at distance *r* from the stimulating probe being activated by a stimulus of amplitude *A* is given by:
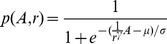
(9)Accordingly, the number of active fibers in the population, *N_pop_*, can be estimated using the following expression:
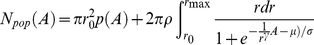
(10)The first term in Equation 10 denotes the number of active afferents located under the stimulating probe, and the second term, those that are away from the probe [13].

### Characterizing the temporal patterning in the PC population response to sinusoids

We have developed a simple integrate-and-fire model that predicts the responses of mechanoreceptive afferents to arbitrary time-varying indentations of the skin with millisecond accuracy [35]. Briefly, the model takes as input the time-varying position of the stimulus and its two derivatives (time-varying speed and acceleration). These inputs are then split into positive and negative going components, to account for the rectification properties of afferents. The outputs of the filters are then summed and constitute the input to an integrate-and-fire model [36]. Using this model, we simulated the response of PC fibers at various distances away from the locus of stimulation. The effective amplitude of the stimulus dropped off as a function of distance according to the (measured) relationship described in Equation 1. The speed of propagation was taken into account by introducing a delay proportional to the distance and inversely proportional to the measured speed at each frequency. Thus, a stimulus at amplitude *A* and frequency *f* propagating at speed *v_f_* impinging upon a Pacinian afferent whose receptive field was distance *r* away from the locus of the stimulation was given by:
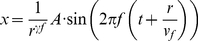
(11)This stimulus was used as input to the transduction model, and the evoked spike times were recorded. We repeated this step for stimuli varying in frequency from 25 to 500 Hz, and for speeds varying from 1 to 20 m/s.

At each frequency and speed, we pooled the spike times and computed a phase histogram, which quantifies the distribution of stimulus phases in which spikes occur. Thus, if spikes have an equal tendency to occur at any phase, the histogram is uniform over the range of possible phases (ranging from 0 to 2π). As a gauge of entrainment, we computed the vector strength from the phase histogram. The vector strength is given by:
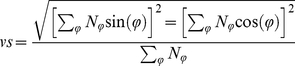
(12)where N_ϕ_ is the number of spikes that occurred in the cycle bin centered on angle ϕ.

### Finite element analysis

Using Abaqus (Dassault Systèmes, Vélizy-Villacoublay, France), we implemented a finite element model to represent the human finger using the cross sectional geometry described by Wu [31]. The 3D model was a simple extrusion of the 2D cross section with an ellipsoidal end. The model included three materials, a bone, the subcutaneous layer, and the skin. No fingernail was included. The material properties were also replica of those described by Wu, including the shear dependence of viscosity in both the cutaneous and subcutaneous layers. Both of these layers also included a “small amount of Rayleigh damping” as described by Wu. A cylindrical probe with a diameter of 2 mm was fixed to the skin at a location analogous to that used in the vibrometry measurements. The probe was set to oscillate with sinusoidal motion at various frequencies (50, 100 200, 300, 500 and 1000 Hz, all at 300 µm) or following a polyharmonic trajectory consisting of seven components (50, 100, 200, 250, 300, 500, and 900 Hz), each at 250 µm. Measurements were made on the skin surface at distances of 1, 2, 4, 8 and 16 mm from the edge of the probe.
